# Performance optimization of interleaved boost converter with ANN supported adaptable stepped-scaled P&O based MPPT for solar powered applications

**DOI:** 10.1038/s41598-024-58852-8

**Published:** 2024-04-06

**Authors:** K. Krishnaram, T. Suresh Padmanabhan, Faisal Alsaif, S. Senthilkumar

**Affiliations:** 1 Department of EEE, E.G.S. Pillay Engineering College, Nagapattinam, Tamil Nadu 611002 India; 2 Department of Electrical Engineering, College of Engineering, King Saud University, 11421 Riyadh, Saudi Arabia; 3 Department of ECE, E.G.S. Pillay Engineering College, Nagapattinam, Tamil Nadu 611002 India

**Keywords:** E-vehicles, Solar system, Interleaved boost converter (ILBC), Maximum power point tracking (MPPT), Artificial neural network supported adaptable stepped-scaled perturb and observe (ANN-ASSPO), Solar energy, Electrical and electronic engineering

## Abstract

Solar energy is the most promising among many renewable energy sources to meet the increasing demand. Photovoltaic (PV) based power generating solutions are expected to gain popularity as a power source for different applications, including independent and grid connected loads, due to their cleanliness, high performance, and high dependability. The efficacy of photovoltaic systems is impacted by several elements, including geographical location, positioning, shadowing effects, and local climate conditions. In order to fulfil the demands of loads, an interleaved boost converter is utilized, which has a reduced number of filters with less stress on the devices. Solar powered systems employ several maximum power point tracking (MPPT) methodologies. However, when there is partial shading, many power peaks arise, which complicates the identification of the overall peak. Although MPPT approaches are designed to measure and maintain the global maximum power point (GMPP), there are still significant oscillations observed around the GMPP with subpar settling time, tracking efficiency, and conversion efficiency. In this work, novel hybrid MPPT technique called artificial neural network supported adaptable stepped-scaled perturb and observe (ANN-ASSPO) method and water cycle optimization based perturb and observe (WCO-PO) have been proposed. Artificial neural network (ANN) has been used to determine the best scaling factor in ANN-ASSPO MPPT. Performance is enhanced in ANN-ASSPO MPPT by using the optimum scaling factor, particularly in situations when the irradiance is rapidly changing/partial shading conditions. Similarly, in WCO-PO MPPT water cycle optimization is used to determine the peak power when the PV panel is subjected to partial shading conditions. The performances of proposed hybrid MPPT ANN-ASSPO and WCO-PO techniques have been compared in terms of power generated, output voltage, average settling time and conversion efficiency. The MATLAB/Simulink tool is employed to carry out the experiment for this study.

## Introduction

The modern, advancing smartphone space seems to have an energy need that is directly proportionate to population. To fulfil this requirement, a lot of fuels are required, but since they are depleting and having negative impacts on the environment via climate change, ozone depletion, and other factors, we must use renewable resources to a greater extent to match requirement and be environmentally friendly. Although there are several renewable energy solutions, solar is among the most often used^1^. Due to its simple maintenance requirements, capacity for productivity, and lack of pollution, PV technology is currently on the rise^2,3^. These systems operate on the straightforward premise of turning sunlight into electricity using the solar cells' internal photovoltaic effect. A photovoltaic cell consists of a fabricated semiconductor layer PN junction^4^. The effectiveness of the PV is influenced by various factors. One of these is geography, or the places they are positioned, the shadowing effect, and the climate there. Other effects are brought on by various PV technical units and the PV array's inclined angle^5^. PV cell output voltages are absolutely insufficient for high-powered applications like solar powered electric vehicles. Difficult switching converters rarely meet the requirement because of their inadequate effectiveness; as a result, soft switching converters were employed as an alternative because of their excellent performance and low degree of loss^6,7^.

Therefore, the voltage can be increased to the required level by using a step-up DC converter. However, employing a standard step-up or boost converter to achieve large voltage output for high-powered applications makes it challenging to preserve greater performance^8,9^. Furthermore, solar powered EV requires a converter with a high-power rating to meet the preferred voltage characteristic because it includes maximum voltage and current ranges. So, the idea of interleaved boost converter (ILBC) is applied. ILBC could be the sole option to accomplish design goals in light of the rising frequency of energy-saving standards. Using a power converter will make it possible to link the solar farm to the grid network (mainly boost converter). Consequently, a direct DC link is established with the PV system. The performance of PV arrays is unaffected, and its main benefits are simplified circuit and cheaper converter expenses. Due to the minimal amount of technology modification required, this architecture may be retrofitted to the present solar based power generation methods^10,11^. With the existing MPPT methods, identification of global peak is difficult with fixed step size. AI techniques have been introduced to overcome the problems associated with fixed step size MPPT technique. Fuzzy logic based variable step size in incremental conductance gave better performance but not in the settling time^12^. The ANN based MPPT gave better solutions to the conventional MPPT but still conversion efficiency and tracking efficiency are poor^13,14^.

Authors devised a method for tracking the MPP in solar PV systems using Reduced Oscillation P&O (ROP&O), aims to minimize the risk of losing track of the MPP direction and to reduce oscillation around the MPP when the solar PV network experiences periodic changes in irradiance^15^. But the settling time is more. Modified variable step size P&O technique aims to achieve the greatest output power from a solar system that is connected to a boost converter^16^. The approach proposed by the authors successfully improved the time it takes to track and reduced the amount of steady state oscillation near the MPP. Recursive bit assignment with neural reference adaptive step (RNA) algorithm proposed by the authors^17^ are not able to track the global peak effectively. The introduction of ANN based hybrid MPPT methods draws more interest from the researchers^18–26^. The advantages of employing ANN include the lack of a need for expertise in precise mathematical models, reduced processing effort, and the capacity to give a clear remedy to multivariable issues. The controller design, necessary input signals, and output signals are three aspects where ANN-based MPPT approaches differ from one another. Authors of^18^ employed ANN as the regulator to boost the effectiveness of the traditional PID controllers; authors of^19,20,24^ applied ANN to calculate MPP.

The majority of the researches^23,24^ used the irradiance and panels’ temperature as the ANN feed when it comes to the needed input signals. But more costly sensors are needed to assess irradiance and panel temperature that could raise the system's total cost. The accuracy and reliability of such detectors are also inadequate. However, the authors of^21^ employed V_oc_ and I_sc_ as the ANN feed, which requires stopping ordinary procedures to measure V_oc_ and I_sc_, which results in power loss. The level of “power variation (dP) and voltage variation (dV)” were then used as the input by them^22^. The “equivalent duty cycle of the MPP (DMPP), the current command value of the MPP (IMPP), the voltage command value of the MPP (VMPP), and the power value of the MPP (PMPP)” are all examples of typical ANN result in the output signals portion. The concern about partial shading conditions with fixed step size conventional MPPT and AI techniques based MPPT brought the researchers to find any potential answers. A hybrid MPPT which integrate ANN and Conventional MPPT could be the solution to address the above said problems. The artificial neural network supported adaptable stepped-scaled PO (ANN-ASSPO) approach is suggested in this paper.

The suggested technique makes use of NN to determine the best scaling factor for the present irradiance level by feeding it the observed voltage and current values of two successive perturbation locations. In-depth simulations are not necessary using the adjustable scaling parameter approach to obtain the optimal scaling factor. Additionally, it can eliminate the challenging calculations necessary when using state estimate techniques for calculation of the irradiation levels. The suggested approach comprises the benefits like easy to use, easy to calculate, and performing at its best under rapidly changing solar irradiance conditions. The suggested approach is contrasted with WCO-PO MPPT to validate the efficiency of the suggested MPPT method. The outcomes of this study show that the robustness and constancy of the suggested method are superior for MPP tracking time. Paper’s organization-second section-methodology; third section-simulation results and discussion; fourth section-conclusion.

## Methodology

The numerical model of PV would be provided in this part. Furthermore, an explanation of the standard PO MPPT will then be given, along with some explanations of how the scaling parameter affects MPPT efficiency. In this proposed work, standalone PV systems is considered.

### Characteristics of solar PV

**Figure 1:**
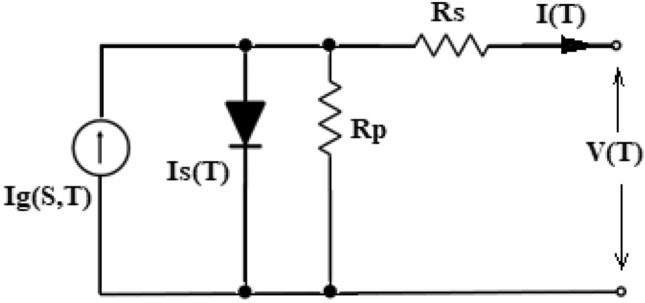
Equivalent circuit model of the solar cell.

The solar cell concept employed in this work is depicted in Fig. 1. Equation (1) indicates the link among both “output voltage and current”.

1 $${\text{I}}_{{\text{T}}} = {\text{I}}_{{\text{g}}} \left( {\text{S,T}} \right) - {\text{I}}_{{\text{s}}} \left( {\text{T}} \right)\left( {{\text{e}}^{{\frac{{{\text{q}}\left( {{\text{R}}_{{\text{S}}} {\text{I}}_{{\text{T}}} {\text{ + V}}_{{\text{T}}} } \right)}}{{{\text{KATN}}}}}} - 1} \right) - \left( {\frac{{{\text{R}}_{{\text{S}}} {\text{I}}_{{\text{T}}} + {\text{V}}_{{\text{T}}} }}{{{\text{R}}_{{\text{P}}} }}} \right)$$

where I_T_ = Load current; V_T_ = Output voltage; I_g_(S,T) = Photoelectric current for S and T; I_s_(T) = Reverse saturation current for particular panel’s temperature; q = Electron charge (1.602 × 10^−19^C); S = Irradiance level; T = Panel’s temperature; N = No. of series-connected cells; K = Boltzmann constant (1.38065 × 10^−23^ J/K); A = Ideality factor of diode; R_S_ = Series resistance; R_P_ = Shunt resistance.

Additionally, I_g_(S, T) and I_s_(T) could be stated as Eqs. (2) and (3), respectively.

2 $${\text{I}}_{{\text{g}}} \left( {\text{S,T}} \right) = \frac{{\text{S}}}{100} \times \left( {{\text{I}}_{{{\text{sc}}}} + \upalpha _{{{\text{I}}_{{{\text{sc}}}} }} \cdot \left( {{\text{T}} - {\text{T}}_{0} } \right)} \right)$$

3 $${\text{I}}_{{\text{S}}} \left( {\text{T}} \right) = {\text{C}}_{0} \cdot {\text{T}}^{3} \cdot {\text{e}}^{{\left( { - \frac{{{\text{E}}_{{\text{g}}} }}{{{\text{KT}}}}} \right)}}$$

C_0_ = Temperature coefficient; I_SC_ = Short circuit current; ${\upalpha }_{{{\text{I}}_{{{\text{SC}}}} }} = {\text{Temperature coefficient of I}}_{{{\text{sc}}}}$; E_g_ = Energy gap.

**Figure 2:**
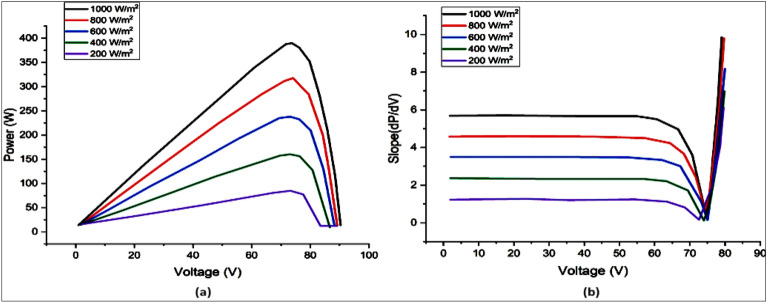
(**a**) PV characteristic and (**b**) slope of PV characteristic.

After establishing the link among both “output voltage and current”, the power output may be calculated by V × I. P–V curve and actual slope ratio for the PV panels are shown in Fig. 2 for various irradiation levels. The differentiation of power to voltage ($${\raise0.7ex\hbox{${{\text{dP}}}$} \!\mathord{\left/ {\vphantom {{{\text{dP}}} {{\text{dV}}}}}\right.\kern-0pt} \!\lower0.7ex\hbox{${{\text{dV}}}$}}$$) is used to determine the P–V curve’s slope.

### Interleaved boost converter (IBC) circuit

**Figure 3:**
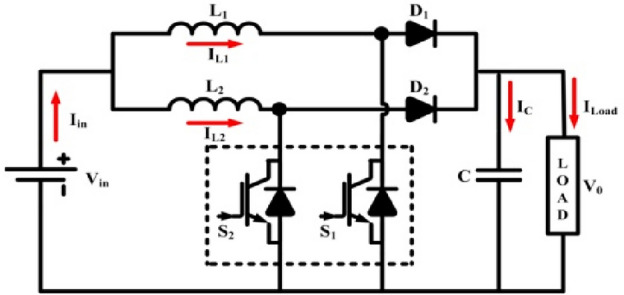
Circuit of 2 phase ILBC.

Since solar cells get limited conversion efficiency, increasing the system performance generally is a crucial design concern for PV systems. Employing highly efficient converters combined with MPPT can help with this to some extent. Such converters must meet two basic requirements: (a) the input current must have no fluctuation, and (b) the performance must be good even at reduced solar intensities. The intermediate converter chops the produced dc voltage and regulates the load's mean dc voltage. Additionally, the converter regularly balances the load's input feature to the PV generator's output feature to get the most power. There have been numerous proposals for intermediate converters with MPPTs for solar panels. At lower-intensity radiations, simple converters like buck and boost converters get to interrupted current mode, which results in inappropriate power device usage and greater conduction losses because of additional current fluctuation. Two phase interleaved DC–DC converter in PV systems has been suggested to lessen input current ripple and to solve the issue of interrupted input current (Fig. 3). The resultant input and output waves have reduced ripple magnitude value. Interleaved functioning also requires less maintenance, improves dependability, and is fault-tolerant. The proposed MPPT is applied to PV systems to extract MPP at all radiations.

**Modes of operation**:

**(i) Mode I:**

**Figure 4:**
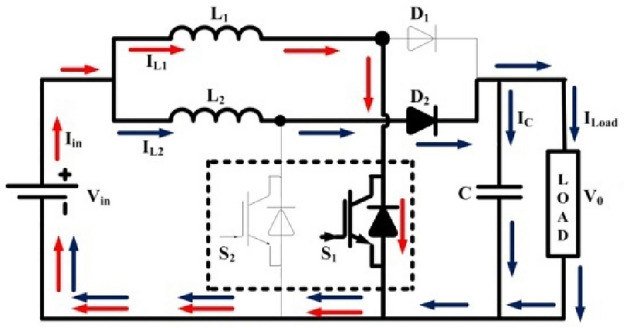
ILBC—Mode I.

At position t = 0; the switch S_1_ is turned on by the gate pulse. The inductor current I_L1_ start rise linearly. During this period the switch S_2_ present in another phase is not gated or not conducting. Therefore, the energy stored previously in the inductor L_2_ passed to the load. The inductors present in two phases namely inductor L_1_ and L_2_. L_1_ starts charging and inductor L_2_ starts discharge to the load through diode. This phase continued till the switch S_1_ is in on state. The mode I operation is shown in Fig. 4.

**(ii) Mode II:**

**Figure 5:**
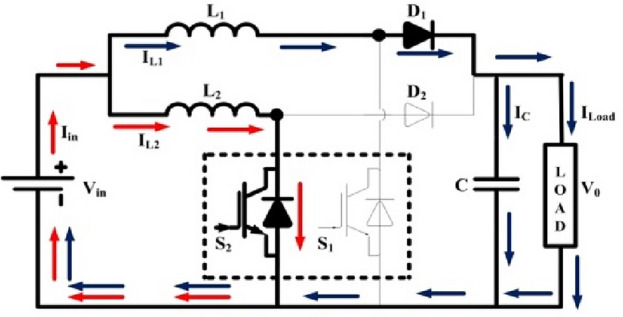
ILBC—Mode II.

At position t = t_1_; the switch S_2_ is turned on and S_1_ is turned off. The energy stored in the inductor L_1_ is transferred to the load through diode. Whereas the inductor L_2_ starts charging. Therefore, the inductor current I_L2_ starts increasing linearly. During this period S_1_ is in off condition. This phase continued till the switch S_2_ is in on state. The mode II operation is shown in Fig. 5.

The output voltage equation of interleaved boost converter is

4 $${\text{V}}_{o} = \frac{{{\text{V}}_{{{\text{in}}}} }}{{\left( {1 - {\text{D}}} \right)}}$$

where Vo & Vin are output and input voltages and D is duty ratio.

**Figure 6:**
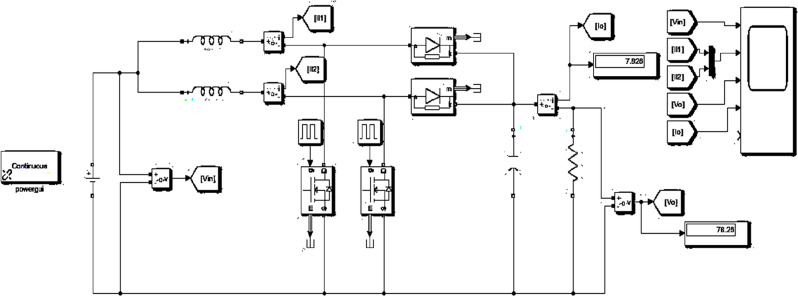
Two phase interleaved boost converter.

Figure 6 shows the Simulink diagram of two-phase interleaved boost converter with R load.

### Principle and motivation for MPPT

**Figure 7:**
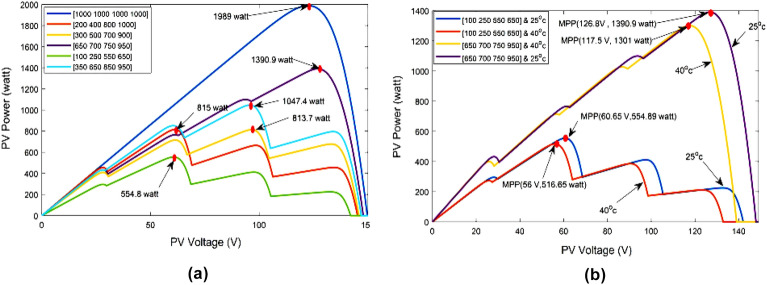
Disparity of MPP with varying levels of temperature and irradiance. (**a**) PV curve under various irradiance with constant temprature. (**b**) PV curve under various irradiance with various tempratures.

Figure 7 shows how incoming temperature and irradiation level, as well as the operating voltage (V) and/or value of the load, affect the power generated by PV systems. The appropriate operating level for a solar array to achieve optimum performance is denoted as MPP. In these situations, the MPPT technique can assist in considerably increasing the power yield of a photovoltaic system by modifying control parameters (load/V) in a manner that guarantees the V will constantly be essentially similar to the optimal V (VMPP). The usage of MPPT is crucial in the solar powered applications because it provides a way to increase power and efficiency despite quickly changing input factors (irradiation level and temperature) based on by the movement of the vehicle and the smooth curves of the PV arrays. Without the requirement for solar panels to be enlarged, which would result in unreasonably increased the system cost and reduced performance, MPPT aids in ensuring large power supply.

### Water cycle approach

The Water Cycle Approach (WCA) concept was drawn from nature and also was depending upon the empirical evidence of the water cycle as well as how streams and rivers naturally migrate towards the sea. Here is a simple explanation of how rivers form and how water flows from mountains to the sea to better comprehend this. A river/stream is formed whenever water moves from one point to a different while moving downhill. This movement of water creates a slope. This implies that the vast number of rivers have their beginnings up in the hills because there is no longer snow where previous glaciers have melted. “Rivers” flow downhill consistently. “Rain and certain other streams” collect water as they descend and eventually end up in the ocean. During this process portion of water of streams or rivers get evaporated. These evaporated water form clouds and return to the ground as rain. These rains create new streams which flow to river or sea. This is a cyclic process. Here sea is assumed as global MPP, streams and rivers are called as local MPP. This WCA approach is used to optimize the PV MPPT^25^.

5 $${\text{C}}_{{\text{i}}} = {\text{Cost}}_{{\text{i}}} = {\text{P}}_{{{\text{FC}}}} = {\text{N}} \times {\text{V}}_{{{\text{cell}}}} \times {\text{I}}_{{{\text{FC}}}}$$

Here C_i_ = function cost.

Rain and evaporation processes come to a stop when

6 $$\left| {{\text{X}}_{{{\text{sea}}}} - {\text{X}}_{{{\text{river}}}} } \right| < {\text{d}}_{{{\text{max}}}}$$

*d*_*max*_ regulates the strength of search close to the sea (best solution).

**Figure 8:**
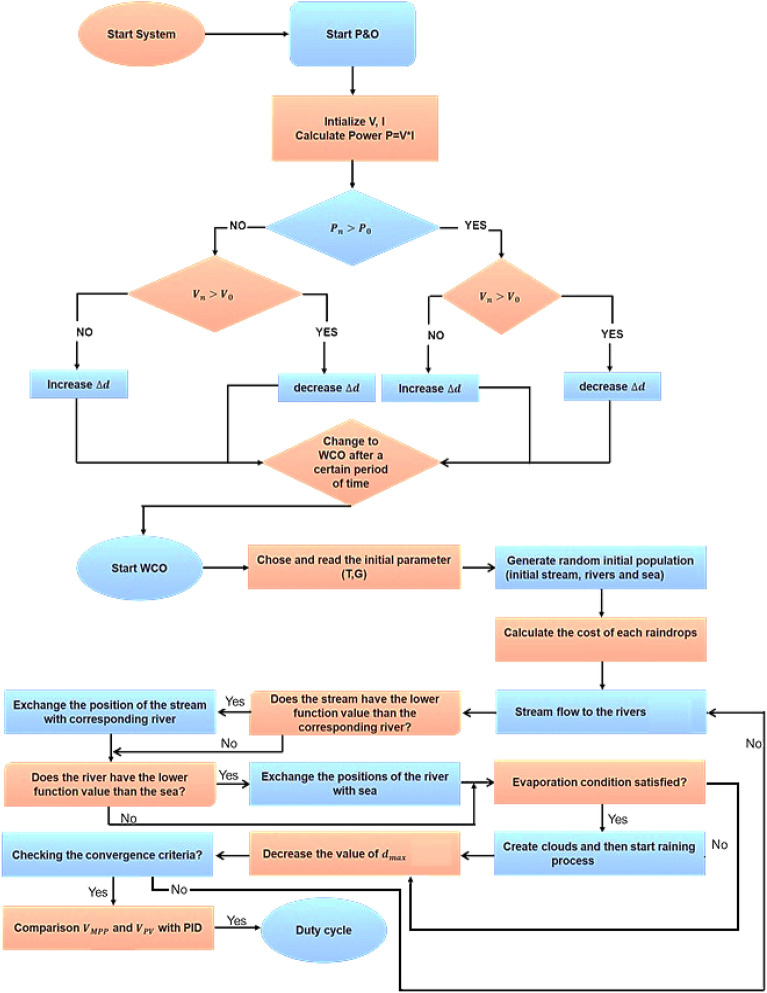
Flowchart of WCO-PO MPPT.

The “raining procedure” is applied while the “evaporation process” is finished. As per it starts to rain, fresh droplets start to produce streams in several places. Fig. 8 portrays the proposed WCO-PO methodology. Equation (7) is employed to point out the fresh positions of the freshly created streams.

7 $${\text{ X}}^{{{\text{new}}}} {\text{stream}} = {\text{X}}_{{{\text{sea}}}} + \sqrt {0.1} \times {\text{randn}}\left( {1,{\text{N}}_{{{\text{var}}}} } \right)$$

### ANN-supported adaptable stepped-scaled PO (ANN-ASSPO)

The precision and monitoring speed requirements are what decide the step size that MPPT methods generally deploy. However, increasing the step length to ramp up monitoring results in a loss in precision, which in turn leads to relatively poor effectiveness, and conversely. An adaptable stepped approach for the ANN-ASSPO MPPT is presented forth in this research with the goal of finding a quick, efficient solution to increase tracking precision and address the problems with conventional MPPT methods. In accordance with the PV array’s operating point, the step size is updated automatically. The step size is updated automatically to the operating point when there is a step change in solar irradiance. The step size is increased while the operational point is omitted to MPP, allowing for quick tracking. The MPPT is accomplished in e-vehicle uses by linking IBC between the PV system and loads. The PV system operating point is used to effectively regulate the IBC's duty cycle relying on a Phase Shift Pulse Width Modulation (PSPWM), which simplifies the control method. The adaptable step size strategy used to solve the aforementioned problem is illustrated as follows:

8 $$D^{*} \left( k \right) = D\left( k \right) - N.\frac{\Delta P}{{\Delta I}}$$

The effectiveness of the MPPT algorithm is modified during the construction process, and N is the scaling parameter. The MPPT incorporates the following features by default:

9 $$\frac{\Delta P}{{\Delta I}} > 0\,PV\,operating\,point\,at\,the\,life\,of\,the\,MPP.$$

10 $$\frac{\Delta P}{{\Delta I}} = 0\,PV\,operating\,point\,at\,MPP.$$

11 $$\frac{\Delta P}{{\Delta I}} = < 0\,PV\,operating\,point\,at\,the\,right\,of\,the\,MPP.$$

The step size must be raised in the first case when the value of $\frac{\Delta P}{{\Delta I}} > 0$ is small, as it will reduce the amount of the phase shift that will be generated. As a result, more PV current will pass through the high-frequency converter, increasing the power that is generated. The third instance, unfortunately, will be the complete reverse. The phase shift remains unchanged if the PV array is operating at the MPP, the value of $\frac{\Delta P}{{\Delta I}} = 0$. This study proposed a more efficient adaptable stepped PO MPPT technique to determine the most adequate scaling factor for the present operating settings, in order to guarantee that the adaptable stepped PO MPPT has the effective tracking performance with maximum accuracy and power failure under various irradiance levels. The structure and use of ANN are also detailed here. In this research, ANN is used to determine the Finest Scaling Parameter (FSP).

**Figure 9:**
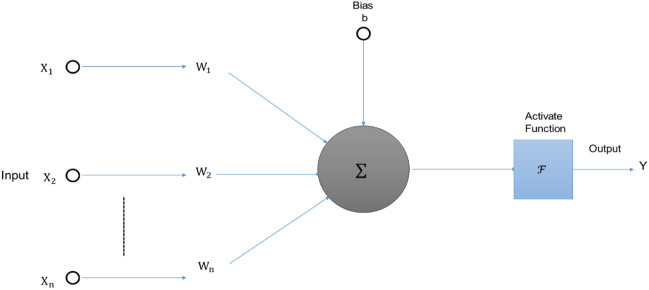
ANN model for FSP calculation.

This study's suggested method first employs ANN to determine the FSP that ought to be employed given the current values of irradiance, and then employs an adaptable stepped PO MPPT approach to detect the MPP. The FSP estimating method used by ANN will be described in this part, along with the process for conducting adaptable stepped PO MPPT using the calculated FSP. A standard ANN conceptual model consists of many neurons, as shown in Fig. 9. NN generates mathematical models by replicating the data analysis of biological NN. This allows NN to replicate behaviour of a system, which are complex and difficult to model. Equation (12) indicates the relationship among both input and output of a neuron; the result is obtained by “multiplying the input by weight (W) and sums”, which is then converted via an “activation function”. Term "NN training" refers to the process of altering weights and bias to produce the desired results. An ANN produces preliminary set of weights among + 1 and − 1 arbitrarily throughout training. The W’s purpose is comparable to the impact of synapsis; when the W is greater, the linked neuron may activate more frequently, and the effect on the network is more noticeable; on the other hand, if the weights are lower, the effect on the NN is less noticeable.

12 $${\text{Y}} = \sum W_{i} X_{i} - b$$

The “activation function” is represented in Eq. (13). The study's training process seems to be the “Levenberg–Marquardt” approach.

13 $$y\left( n \right) = \frac{{e^{n} - e^{ - n} }}{{e^{n} + e^{ - n} }} = Tansig \left( n \right)$$

**Figure 10:**
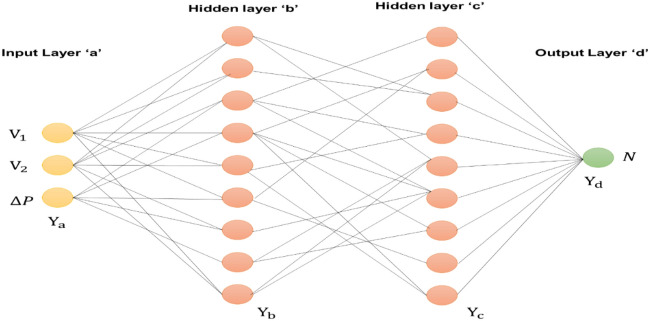
Artificial neural network architecture.

**Figure 11:**
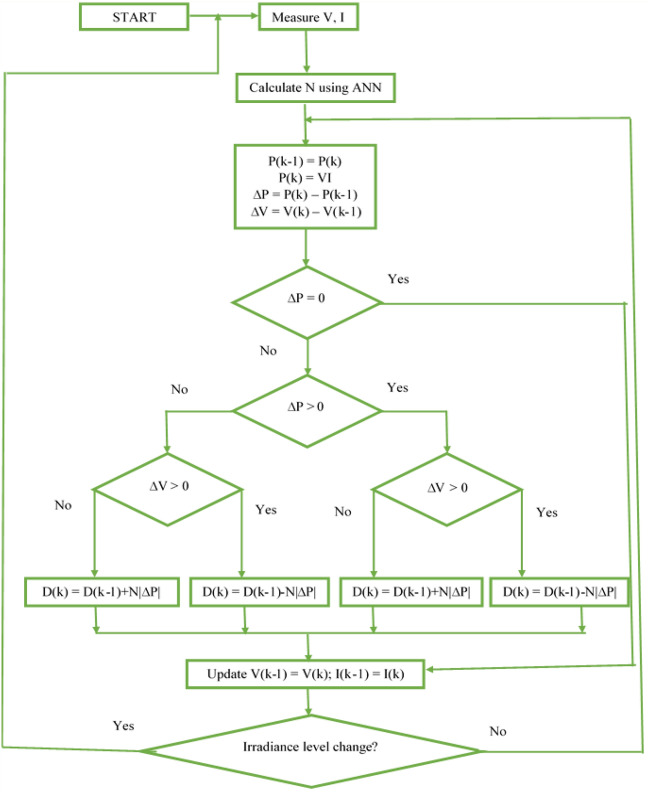
Flow of ANN-ASSPO MPPT.

The back-propagation NN used in this research has 3 layers: an input layer (IL), a hidden layer (HL), and an output layer (OL) shown in Fig. 10. The amount of IL’s input neurons varies depending on the complexity of the problems being addressed. The IL and OL are separated by a layer of neurons called the HL, which serves as a representation of the nonlinear relationship between the two. There’s presently no ordinary procedure to identify the HL’s setting; a superior set-point is typically achieved via many experiments. Instead, HL's setting must be determined depending upon the problems’ difficulty. The backpropagation algorithm used in this method initiated with random weights. The output is calculated when the input data are fed to the network during the forward path. Once the error is calculated, network weights will be adjusted according to the error calculated in the backward path. The network is fed with measured PV power, voltage and current as inputs and scaling factor (N) is an output which will be calculated by the network. The error in the output will be calculated from obtained scaling factor and target scaling factor to identify the global peak in PV characteristics. The aim is to adjust the weights using gradient descent algorithm to reduce the scaling factor error to identify the global peak until the network learns the training data. PV’s power, voltage and current are inputs to this network. The finest scaling parameter N is available in the output layer. This scaling parameter is used in P&O method to generate maximum power even under partial shading conditions. Fig. 9 depicts the ANN architecture for FSP calculation. Here, the output is FSP. Fig. 11 illustrates the ANN-ASSPO MPPT’s procedure. The benefits of the traditional PO MPPT are “High tracking speed, high precision, low tracking loss, and etc.” An ANN-ASSPO MPPT algorithm was presented in this study because the constant scaling factor causes its performance to considerably drop whenever the irradiation level varies.

### Ethical approval

This paper does not contain any studies with human participants or animals performed by any of the authors.

## Simulated results and discussion

**Figure 12:**
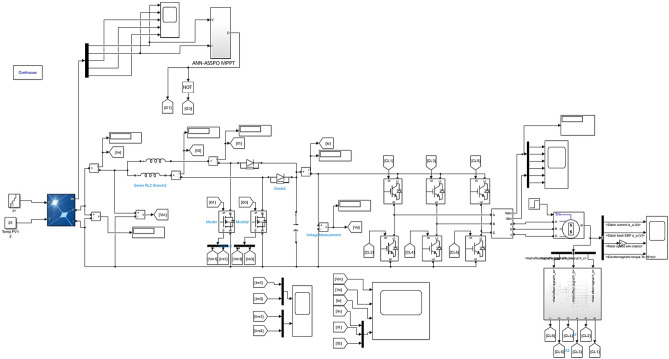
Solar fed two phase IBC with BLDC drive.

The suggested ANN-ASSPO MPPT's power extracting abilities and consistency are compared with WCO-PO in an effort to assess its ability to function in comparison to SPEVs. With the MATLAB/Simulink application, the modelling of a SPEV is performed with an ANN and ASSPO under various climatic circumstances have been crucial to the motion of a SPEV, i.e., with rapid variations in temperature and irradiance levels. Tables 1 and 2 shows the simulation parameters of IBC and electrical characteristics of PV panel from Applied Materials respectively. Table 3 shows the irradiance and temperature variations assumed during simulations. The implementation of ANN-ASSPO based MPPT with two phase interleaved boost converter has been realized under MATLAB environment. The circuit implemented in MATLAB has been shown in Fig. 12.

**Table 1:** Two phase ILBC simulation parameters.

Parameters	Values
*D*	50%
*f*_*SW*_	20 kHz
*R*	10 Ω
*L*_1_, *L*_2_	120 µH
*C*	300

**Table 2:** PV’s specifications.

V_oc_ = open circuit voltage	280 V
I_sc_ = short circuit current	2.6 A
P_max_ = maximum power	458 W
I_MPP_ = operating current	2.16 A
V_MPP_ = operating voltage	216 V
Temperature constant of *V*_*oc*_	− (0.401) %/°C
Temperature constant of *I*_*sc*_	(0.104) %/°C

**Table 3:** Various cases with irradiance and temperature levels.

Cases	W/m^2^	°C
Case-1: Rapid rise in irradiance	100–1100	25
Case-2: Rapid reduction in irradiance	1000–400	25
Case-3: Rapid rise in temperature	1000	10–50
Case-4: Rapid reduction in temperature	1000	60–50

### Case-1: Rapid rise in irradiance level

**Figure 13:**
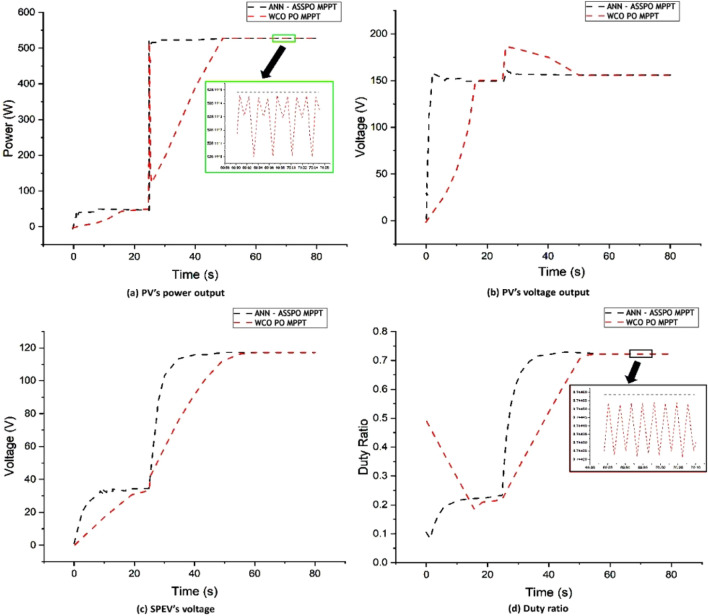
Case-1 results of ANN-ASSPO & WCO-PO MPPT methods.

Firstly, the effectiveness of the proposed ANN-ASSPO and existing MPPT techniques was compared for a constant temperature of 25 °C and an instant rise in irradiation between 100 and 1100 W/m^2^ with t = 25 s. As indicated in Fig. 13, we find that the captured power grew along with the sharp rise in irradiation. We observe a highly abrupt overshoot of the power level in WCO-PO MPPT case. The power indication for the WCO-PO MPPT grows quickly, lowers suddenly, and afterwards gradually builds to a peak point that appears to be constant. Zoomed in, we notice that the WCO-PO MPPT produced waveform is essentially unsteady and fluctuates around a mean. In the application of the WCO-PO MPPT, identical overshoots are produced for the voltage levels. This not only result in less energy getting collected, but also it causes frequent, repeated electric shifts to the components, possibly limiting their lifetime. We observe that the suggested MPPT performs significantly better. Firstly, the steady state output is quite stable for voltage and power. In addition, we see that contrasted to the WCO-PO MPPT with reduced overshoot, the reaction time to identify MPP and accomplish peak power extraction is lower. The values of output power variation, output voltage variation, duty ratio changes and average settling time under Case-1 are given in Table 4.

**Table 4:** Performance of ANN-ASSPO & WCO-PO MPPT when irradiance changes from 100 to 1100 W/m^2^.

@25°C	PV’s power output (W)		PV’s output voltage (V)		Duty ratio		Avg. settling time (s)	
MPPT	100 W/m^2^	1100 W/m^2^	100 W/m^2^	1100 W/m^2^	100 W/m^2^	1100 W/m^2^	100 W/m^2^	1100 W/m^2^
ANN-ASSPO	51.57	511.76	146.49	158.78	0.22	0.72	3.76	2.28
WCO-PO	49.85	506.83	144.48	154.37	0.21	0.72	17.59	24.48

### Case-2: Rapid reduction in irradiance

**Figure 14:**
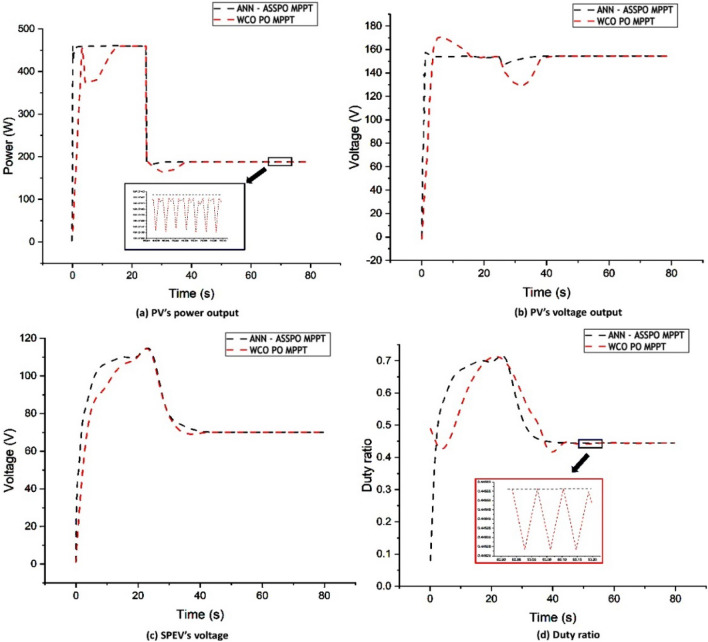
Case-2 results of ANN-ASSPO & WCO-PO MPPT methods.

In this scenario as seen in Fig. 14, we contrast the suggested MPPT's effectiveness with that of the existing WCO-PO MPPT with a constant temperature of 25 degree Celsius and a nearly instantaneous fall in irradiation between 1000 and 400 W/m^2^ during t = 25 s. Comparable results are noted for the suggested MPPT's reduced time responsiveness for power stabilizing and identifying the peak power. The difference in performance among the two systems is, however, lower than it was in the earlier case of the sharp rise in irradiation. The main benefit of the suggested MPPT in this situation is during stable operation with increased voltage and power reliability and decreased overshoot. Under Case-2, the output power, output voltage, duty ratio variation and average settling time are given in Table 5.

**Table 5:** Performance of ANN-ASSPO & WCO-PO MPPT when irradiance changes from 1000 to 400 W/m^2^.

@25°C	PV’s power output (W)		PV’s output voltage (V)		Duty ratio		Avg. settling time (s)	
MPPT	1000 W/m^2^	400 W/m^2^	1000 W/m^2^	400 W/m^2^	1000 W/m^2^	400 W/m^2^	1000 W/m^2^	400 W/m^2^
ANN-ASSPO	457.67	185.95	153.88	151.56	0.71	0.45	1.52	4.08
WCO-PO	455.57	183.48	152.76	149.87	0.71	0.45	16.06	15.3

### Case-3: Rapid rise in temperature level

**Figure 15:**
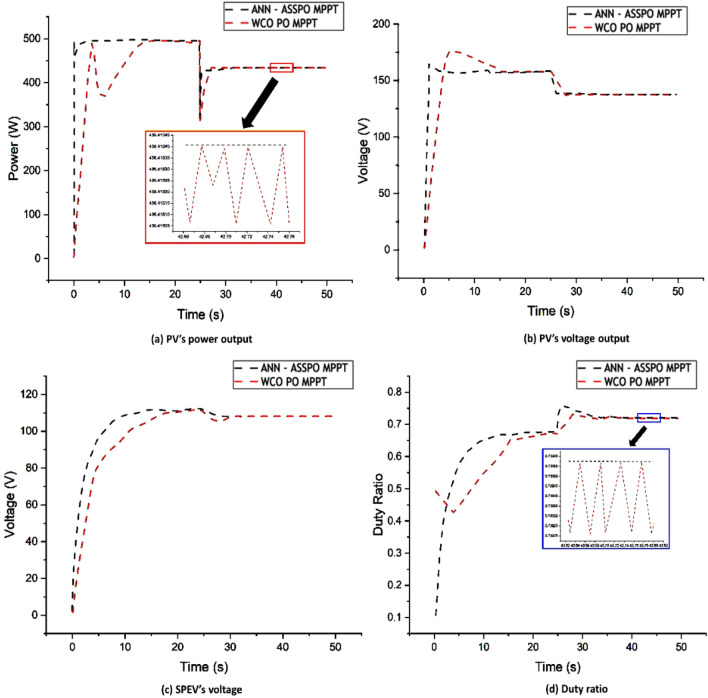
Case-3 results of ANN-ASSPO & WCO-PO MPPT methods.

It is crucial to research how the two controllers react to temperature fluctuations since temperature has a harmful effect on how effective PV systems perform. At a constant irradiation of 1000 W/m^2^, an instantaneous temperature rise between 10 and 50 °C would be applied to both MPPT techniques (as seen in Fig. 15). With a faster reaction time, reduced overshoot, and far less fluctuation in steady-state condition, the suggested MPPT's abilities are obviously more noticeable. The output power, output voltage, duty ratio changes and average settling time when temperature rise between 10 and 50 °C are given in Table 6.

**Table 6:** Performance of ANN-ASSPO & WCO-PO MPPT when temperature changes from 10 to 50 °C.

@1000 W/m^2^	PV’s power output (W)		PV’s output voltage (V)		Duty ratio		Avg. settling time (s)	
MPPT	10°C	50°C	10°C	50°C	10°C	50°C	10°C	50°C
ANN-ASSPO	483.48	424.37	156.66	133.33	0.67	0.71	2.4	1.6
WCO-PO	479.57	421.49	154.25	130.12	0.64	0.68	14.8	2.8

### Case-4: Rapid reduction in temperature level

**Figure 16:**
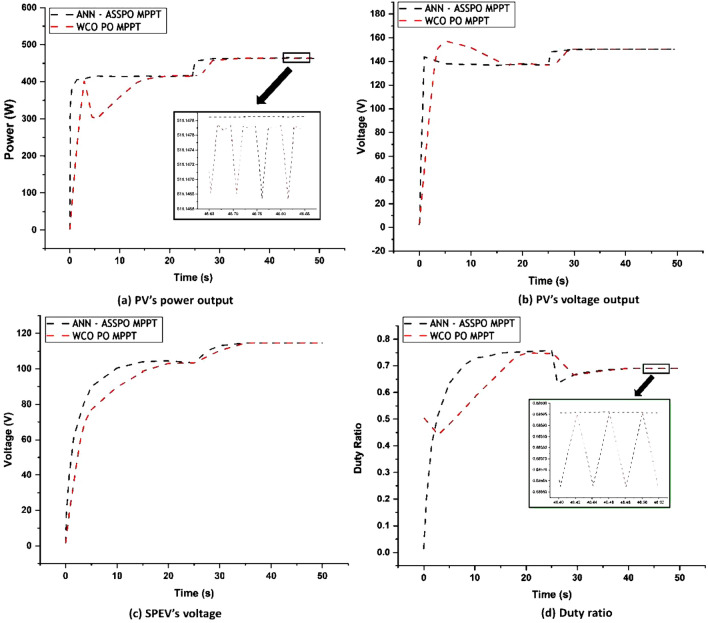
Case-4 results of ANN-ASSPO & WCO-PO MPPT methods.

As seen in Fig. 16, the same results as indicated above are observed in which the temperature quickly drops from 60 to 50 °C with a constant irradiation of 1000 W/m^2^, but with improved time of responsiveness, minimal overshoot, and less fluctuations in the stable condition. For any solar powered applications, when rapid fluctuations in temperature and irradiation are constant, this condition provides a valuable benefit in time and energy. The values of output power changes, output voltage changes, duty ratio changes and average settling time under Case-4 are given in Table 7.

**Table 7:** Performance of ANN-ASSPO & WCO-PO MPPT when temperature changes from 60 to 50 °C.

@1000 W/m^2^	PV’s power output (W)		PV’s output voltage (V)		Duty ratio		Avg. settling time (s)	
MPPT	60°C	50°C	60°C	50°C	60°C	50°C	60°C	50°C
ANN-ASSPO	415.49	448.36	138.32	145.99	0.75	0.70	4.0	1.2
WCO-PO	413.52	445.78	135.76	142.86	0.74	0.70	14.6	4.6

**Figure 17:**
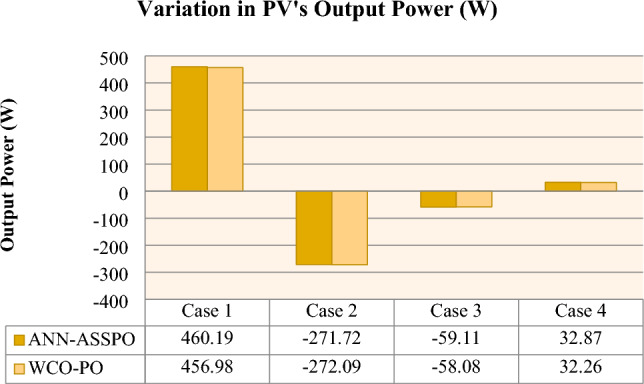
Variation of Output Power in all four cases.

**Figure 18:**
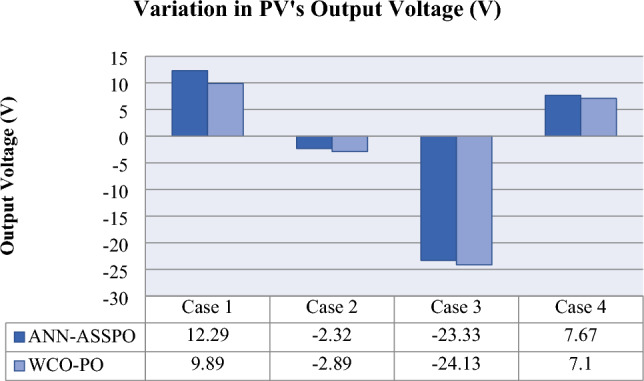
Variation of Output Voltage in all four cases.

**Figure 19:**
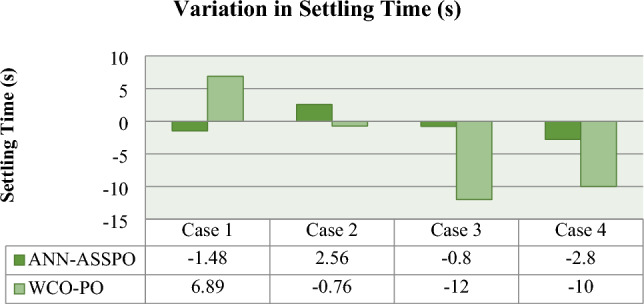
Variation of settling time in all four cases.

From Fig. 17, it can be observed that the ANN-ASSPO achieves the highest power output. The output voltage of PV panel is more in ANN-ASSPO MPPT than WCO-PO MPPT which was shown in the Fig. 18. The settling time is very less in ANN-ASSPO MPPT than WCO-PO MPPT in three cases except case II. When there is sudden decrease in irradiance from 1000 to 400 W/m^2^, ANN-ASSPO MPPT takes more time to settle. The variations in settling time of both MPPT techniques in all four cases are shown in Fig. 19. The values of PV’s output power, output voltage, duty ratio and conversion efficiencies of ANN-ASSPO MPPT and WCO-PO MPPT are shown in Table 8.

**Table 8:** Overall performance of ANN-ASSPO & WCO-PO MPPT in all four cases.

MPPT	PV’s power output (W)		PV’s output voltage (V)		Duty ratio		Avg. settling time (s)		Conversion efficiency	
	100 W/m^2^	1100 W/m^2^	100 W/m^2^	1100 W/m^2^	100 W/m^2^	1100 W/m^2^	100 W/m^2^	1100 W/m^2^	100 W/m^2^	1100 W/m^2^
Case I @25°C										
ANN-ASSPO	51.57	511.8	146.49	158.8	0.22	0.72	3.76	2.28	11.3	99.61
WCO-PO	49.85	506.8	144.48	154.4	0.21	0.72	17.59	24.5	10.9	98.64

	1000 W/m^2^	400 W/m^2^	1000 W/m^2^	400 W/m^2^	1000 W/m^2^	400 W/m^2^	1000 W/m^2^	400 W/m^2^	1000 W/m^2^	400 W/m^2^
Case II @25°C										
ANN-ASSPO	457.67	186	153.88	151.6	0.71	0.45	1.52	4.08	99.9	40.6
WCO-PO	455.57	183.5	152.76	149.9	0.71	0.45	16.06	15.3	99.5	40.06

	10°C	50°C	10°C	50°C	10°C	50°C	10°C	50°C	10°C	50°C
Case III @1000 W/m^2^										
ANN-ASSPO	483.48	424.4	156.66	133.3	0.67	0.71	2.4	1.6	99.9	92.66
WCO-PO	479.57	421.5	154.25	130.1	0.64	0.68	14.8	2.8	99.1	92.03

	60°C	50°C	60°C	50°C	60°C	50°C	60°C	50°C	60°C	50°C
Case IV @1000 W/m^2^										
ANN-ASSPO	415.49	448.4	138.32	146	0.75	0.7	4	1.2	90.7	97.9
WCO-PO	413.52	445.8	135.76	142.9	0.74	0.7	14.6	4.6	90.3	97.33

## Conclusion

This work proposed a novel ANN-supported adaptable stepped-scaled PO (ANN-ASSPO) MPPT approach for optimizing the performance of PV system based standalone applications. The suggested NN was used to acquire the FSP and enhance the efficiency of the ASSPO MPPT approach. The problem with WCO-PO MPPT has been highlighted, and the suggested solution was used to overcome it. The MATLAB/SIMULINK toolset was used to produce the research's findings. The simulation findings demonstrate the viability and efficacy of the suggested approach. The simulations revealed that rapidly adjusting factors that affect power output were necessary to obtain the most notable disparities. Additionally, the suggested ANN-ASSPO has reduced oscillations around global peak because of adaptable stepped scale with average conversion efficiency of 99.80% and average settling time of 2.06 s. Therefore, ANN-ASSPO based MPPT outperforms the WCO-PO MPPT in all above-mentioned terms. Power fluctuation reduction benefits greatly from the ANN-ASSPO MPPT's due to its stability.

## Data Availability

The datasets used and/or analysed during the current study are available from the corresponding author upon reasonable request.
